# Six degree-of-freedom knee joint kinematics in obese individuals with knee pain during gait

**DOI:** 10.1371/journal.pone.0174663

**Published:** 2017-03-24

**Authors:** Jing-Sheng Li, Tsung-Yuan Tsai, David T. Felson, Guoan Li, Cara L. Lewis

**Affiliations:** 1 Bioengineering Laboratory, Department of Orthopaedic Surgery, Massachusetts General Hospital / Harvard Medical School, Boston, Massachusetts, United States of America; 2 College of Health and Rehabilitation Science: Sargent College, Boston University, Boston, Massachusetts, United States of America; 3 Clinical Epidemiology Research and Training Unit, Boston University School of Medicine, Boston, Massachusetts, United States of America; 4 NIHR Manchester Musculoskeletal Biomedical Research Unit, Manchester Academic Health Science Centre, Manchester, United Kingdom; University of Memphis, UNITED STATES

## Abstract

Knee joint pain is a common symptom in obese individuals and walking is often prescribed as part of management programs. Past studies in obese individuals have focused on standing alignment and kinematics in the sagittal and coronal planes. Investigation of 6 degree-of-freedom (6DOF) knee joint kinematics during standing and gait is important to thoroughly understand knee function in obese individuals with knee pain. This study aimed to investigate the 6DOF knee joint kinematics in standing and during gait in obese patients using a validated fluoroscopic imaging system. Ten individuals with obesity and knee pain were recruited. While standing, the knee was in 7.4±6.3°of hyperextension, 2.8±3.3° of abduction and 5.6±7.3° of external rotation. The femoral center was located 0.7±3.1mm anterior and 5.1±1.5mm medial to the tibial center. During treadmill gait, the sagittal plane motion, i.e., flexion/extension and anterior-posterior translation, showed a clear pattern. Specifically, obese individuals with knee pain maintained the knee in more flexion and more anterior tibial translation during most of the stance phase of the gait cycle and had a reduced total range of knee flexion when compared to a healthy non-obese group. In conclusion, obese individuals with knee pain used hyperextension knee posture while standing, but maintained the knee in more flexion during gait with reduced overall range of motion in the 6DOF analysis.

## Introduction

The prevalence of obesity is increasing in the United States and throughout the world [1, 2]. In 2011–2012, 34.9% of adults in the United States were obese [3]. Musculoskeletal disorders are commonly seen in obese individuals and one of the most common and disabling of these is knee osteoarthritis (OA) [4].

Individuals who are obese and have knee pain may adopt different gait patterns to compensate both for the extra weight and joint pain. While no prior studies have focused specifically on obese persons with knee pain, studies of obese adults showed that they walked with decreased velocity [5, 6]. As body mass index (BMI) increases, gait speed decreases [7]. Past studies of knee joint kinematics mainly focused on sagittal and coronal plane motions, i.e., knee joint flexion/extension [5, 8–10] and varus/valgus rotation [9, 10]. Even so, there is no clear consensus on knee joint kinematics in obese individuals during walking. For example, Haight et al.[8] reported that obese individuals walked with a less flexed knee during the stance phase compared to non-obese individuals. Vismara et al. [11] concluded that the range of knee flexion excursion during gait was not significantly different than a healthy group. The inconsistency and variation in the literature may be due to differences in measurement methods or the presence of different lower extremity joint pathology such as pain which is extremely common in obese adults and may cause gait modifications. For instance, knee pain is a major symptom in individuals with knee OA and reduced range of knee flexion during gait has been frequently reported [12–14].

Most previous investigations of obese gait used skin marker motion analysis systems [5, 6, 8, 15]. The kinematic data derived from a skin marker-based motion capture system are vulnerable to soft tissue artifacts [16, 17]. According to Peters et al.[16] the magnitudes of soft tissue artifacts were greater than 30 mm on the femur and up to 15 mm on the tibia. Cappozo et al. [18] quantified rotation errors of 6–20° on the femur and 4–10° on the tibia [18]. Although some biomechanical researchers have tried to reduce soft tissue artifact by using an obesity-specific marker set to investigate gait patterns [15], accurate detailed kinematics in 6 degree-of-freedom (6DOF) among obese individuals have not been elucidated. As past studies have shown that standing posture and gait pattern were affected by body weight [5, 8–10, 19], a better understanding of the standing posture and gait pattern in 6DOF is critical. Such information would provide a foundation on which to build better walking programs, which are commonly suggested as a means to increase the energy expenditure of obese individuals [20, 21] and especially to design a treatment program for obese persons with knee pain.

Recent advancement of imaging technology, such as the dual fluoroscopy imaging system (DFIS), makes it possible to track in-vivo bone motion without soft tissue artifacts [22–24]. This methodology has been validated[25] with submillimeter and subdegree accuracy in translation and rotation. In this study, we evaluated knee joint kinematics in standing and during gait in obese individuals with knee pain using DFIS[25] and compared them to a healthy non-obese control group without knee pain. We hypothesized that there would be distinct motion patterns in obese individuals with knee pain which would differ from typical patterns in a healthy population.

## Methods

### Participants

Ten obese individuals with knee pain on most of the last 30 days (8 females, 2 males; age (mean±SD): 42.8±10.1 (range: 30.2–56.5) years; BMI: 39.6±2.8 (range: 35.3–44.3) kg/m^2^, body mass: 109.6±13.0 (range: 86.0–130.9) kg, body height: 166.1±8.5 (range: 152–184) cm) were recruited to participate in this study and the study protocol was approved by Boston University School of Medicine and Massachusetts General Hospital Institutional Review Boards. Written informed consent approved by both Institutional Review Boards was obtained for each subject. All participants reported being able to walk without assistance. For each participant, the more painful knee was selected for evaluation except that knees that had undergone surgery were excluded. If knee pain increased sharply in a short period of walking, the participant was also excluded.

### Experiment procedures

A posteroanterior standing knee joint plain radiograph in a slight flexion position was taken for each participant in the obesity group and graded by an experienced rheumatologist (DTF) to determine the severity of osteoarthritis using Kellgren-Lawrence grading scale[26]. The selected knee was then scanned by a 3-Tesla MR machine (Philips, Achieva, Eindhoven, The Netherlands) with a sagittal Proton Density-Weighted (PDW), Spectral Attenuated Inversion Recovery (SPAIR) sequence (FOV: 160mm x 160mm, TR = 1800ms, TE = 30ms, flip angle = 90°, thickness = 1mm, in-plane resolution = 512 x 512). All MR images were reviewed and used for segmentation to construct a 3-dimensional (3D) bony surface model of the knee, including the femur and tibia. To better understand the knee joint pain status, we adopted two relevant questions, one from the Western Ontario and McMaster Universities Osteoarthritis Index (WOMAC) questionnaire (pain on 4-point Likert scale while walking on a flat surface) and one on overall pain level using a visual analog scale. The Likert scale was scored 0 as no pain to 4 as extreme pain and visual analog scale was rated on a 0–100 scale.

**Fig 1:**
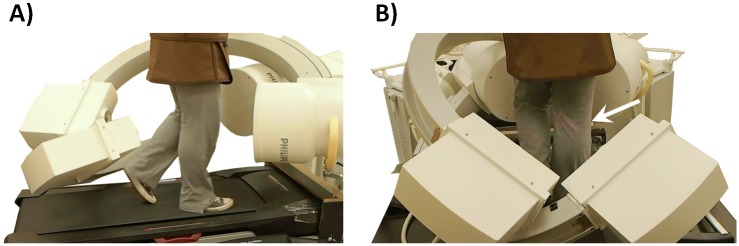
A) Treadmill gait with dual fluoroscopic imaging system setup and protection bar setup. B) recording the treadmill gait on the index knee (arrow).

**Fig 2:**
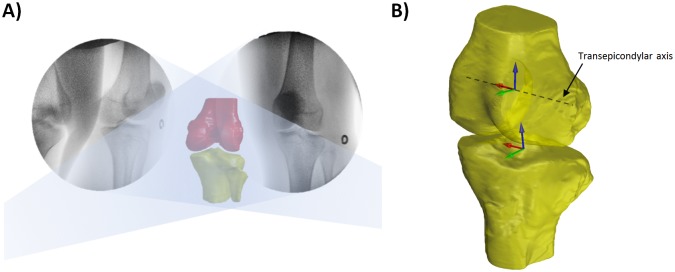
A) The MR-based 3D knee bony models were matched to the silhouettes of the corresponding bones in the fluoroscopic images. B) Illustration of coordinate systems for the femur and tibia.

In the fluoroscopic experiment, one pair of dual fluoroscopic images of the knee in static standing was obtained to evaluate comfortable standing posture in the participants with obesity. The participant was then asked to walk on a treadmill at 1.5mph (0.67m/s) with a thyroid collar over their throat and lead apron over their chest to upper thigh. After a warm-up period on the treadmill, the knee was imaged by the DFIS (Philips, BV Pulsera, Eindhoven, The Netherlands) at 30 frames per second with an 8ms pulse-width (Fig 1A) [22, 25]. This system captured knee motion along two oblique views (medioposterior-lateroanterior and lateroposterior-medioanterior) (Fig 1B). All the output images were corrected for distortion using a calibration grid and customized algorithm [27, 28] developed on MATLAB software (Mathwork, Natick, MA, USA). The fluoroscopic images and the MR-based 3D knee bony models were then imported to a virtual fluoroscopic environment for 2D-3D registration procedure [24], where the projection of the 3D knee model was matched to the 2D silhouette of the corresponding bones in the fluoroscopic images (Fig 2A). The knee joint motion during the gait cycle was represented by a series of knee joint models.

### Data analysis

To calculate the kinematics during the stance phase of gait, we built a coordinate system for each femur and tibia. The coordinate system was used in both the obese with knee pain group and the healthy non-obese group to present the subject-specific data (Fig 2B). In the femoral coordinate system, we first defined the transepicondylar axis (TEA) and long axis of the distal femur [22]. The TEA was defined as the medial-lateral axis and the mid-point of the axis as the femoral center. The cross product of the TEA and the long axis was the anterior-posterior axis. For the tibial coordinate system, two circles were created to fit the medial and lateral plateaus separately [22]. The line connecting the centers of these two circles was defined as the medial-lateral axis and the mid-point as the tibial center. The cross product of the medial-lateral axis and the proximal tibial long axis was the anterior-posterior axis of the tibia.

The knee rotation angles were calculated using an Cardan angle sequence (Flexion/Extension, Adduction/Abduction, Internal/External rotation) [29]. The knee translations were represented along the anterior-posterior, medial-lateral, and superior-inferior directions of the tibia. Typically unfiltered data from two stance phases of the gait cycles were analyzed and the data points of the two trials were averaged to represent the motion data for each participant. The 6DOF kinematic data of the knee joint were normalized to the stance phase of the gait cycle and averaged among all participants. The 6DOF range of motion was calculated as the maximum value minus the minimum value during the stance phase of the gait cycle.

### Control group

Previously published 6DOF knee kinematic data during gait from non-obese participants without knee pain or previous surgery provided a control group for comparison [22]. These data were collected in the same lab using the same fluoroscopic imaging technique and walking speed as the current study. The demographics of the control participants were: 6 males and 2 females, aged 32–49 years, mean BMI 23.5kg/m, 5 left knees and 3 right knees.

### Statistical analysis

Independent t-tests were used to test for differences between the group of obese individuals and group of healthy controls at specific events of interest during the stance phase of the gait cycle. The dependent variables included: flexion-extension, adduction-abduction, internal-external rotation, anterior-posterior, medial-lateral, and superior inferior translations at heel strike, end of loading response, end of mid-stance, end of terminal stance and toe-off. The level of significance was set at 0.05 two sided.

## Results

### Radiograph findings

The right knee was evaluated in 5 of the 10 participants with obesity, and the left knee in the other 5. Eight of the 10 had medial knee pain. The mean Kellgren-Lawrence scale of the obese individuals with knee pain was 1.2±0.8 (Table 1). Four out of the 10 were considered to have radiographic OA based on Kellgren-Lawrence grade ≥ 2 [26]. The WOMAC scores while walking were 1.9±1.3 out of 4 and the visual analog scale for knee pain was 66.2±16.7 out of 100 (Table 1).

**Table 1:** Side of index knee, the knee pain compartment, X-ray findings, and WOMAC scores.

Participant	Side of index knee	Knee pain compartment	Kellgren-Lawrence scale	WOMAC Walking	WOMAC Pain
# 1	Left	Medial	1	4	50
# 2	Left	Medial	2	3	90
# 3	Right	Medial	1	1	41
# 4	Left	Lateral	2	1	60
# 5	Right	Medial	0	2	70
# 6	Right	Lateral	2	0	70
# 7	Right	Medial	2	3	90
# 8	Left	Medial	0	2	70
# 9	Left	Medial	1	–	–
# 10	Right	Medial	1	1	55
Average			1.2	1.9	66.2
SD			0.8	1.3	16.7
Maximum			2	4	90
Minimum			0	0	41

“ – ” indicates missing value

### 6 degrees of freedom knee kinematics in comfortable standing posture

In the 10 individuals with obesity, the standing knee position was in 7.4±6.3° of extension, 2.8±3.3° of abduction, and 5.6±7.3° of external rotation. The femoral positions with respect to the tibia axes were 5.1±1.5 mm, 0.7±3.1 mm, and 29.5±1.8 mm along the medial-lateral, anterior-posterior, and superior-inferior directions, respectively (Table 2).

**Table 2:** Standing posture of the knee joint in 6 degrees of freedom.

Participant	Flexion (+)/extension (-)	Adduction(+)/abduction (-)	Internal (+)/external (-) tibial Rotation	Medial(+)–lateral (-) direction	Anterior (+)–posterior (-) direction	Superior(+)–inferior (-) direction
# 1	-1.9	-7.8	-16.8	7.3	3.6	29.4
# 2	-6.8	-8.1	-14.7	4.3	5.9	27.7
# 3	-5.2	1.0	-5.5	5.6	-1.5	32.5
# 4	-20.2	1.3	-10.3	2.1	2.0	29.6
# 5	-12.4	-1.7	-1.5	4.6	1.5	28.8
# 6	-5.6	-3.9	7.9	4.4	0.7	28.8
# 7	3.0	-3.4	-2.7	6.9	-3.1	26.4
# 8	-6.8	-1.5	-0.7	6.0	1.0	29.7
# 9	-12.2	0.0	-8.4	5.0	1.1	32.1
# 10	-6.0	-4.0	-3.2	4.7	-4.5	30.0
Average	-7.4	-2.8	-5.6	5.1	0.7	29.5
SD	6.3	3.3	7.3	1.5	3.1	1.8
Maximum	3.0	1.3	7.9	7.3	5.9	32.5
Minimum	-20.2	-8.1	-16.8	-2.1	-3.1	26.4

### Spatiotemporal parameters and the 6 degrees of freedom knee kinematics during gait

The average stride length in the obese group was 86.8±9.2cm and the average cadence was 93.0±10.4 steps/min. The duration of the stance phase was 0.80±0.07 second.

**Fig 3:**
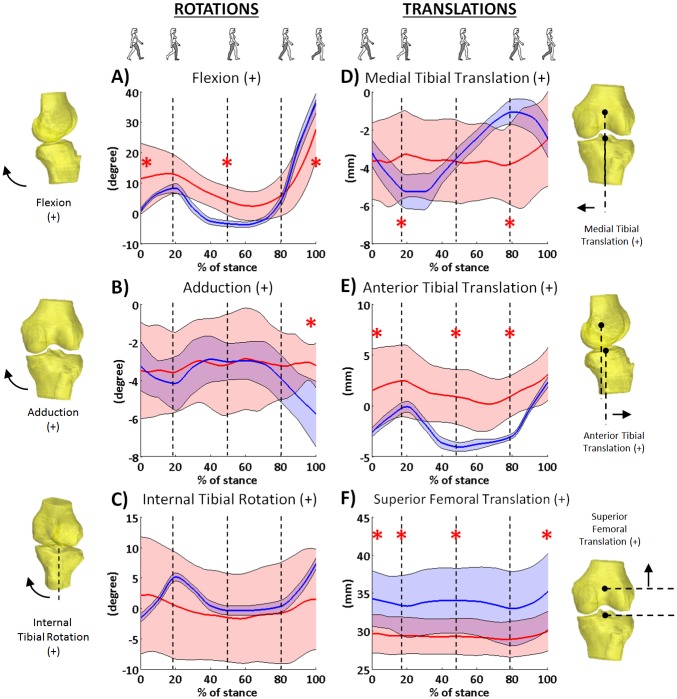
Six degree-of-freedom kinematics during stance phase of treadmill gait in obese individuals with knee pain (red. S1 File) and a healthy population (blue, data previously published in Kozanek et al. J Biomech. 2009;42(12):1877–1884.). Solid line indicates the mean and shade area for ±1SD. Asterisk denotes significant difference between two groups.

The obese group walked with increased flexion in the first half of the stance phase, and with reduced flexion in the preswing phase compared to the non-obese group. Specifically, the obese knees started in a more flexed position at initial contact (11.4±11.7° vs. 0.9±0.7°, *p* = 0.02), and at the end of the mid-stance phase (4.0±4.7° vs. -3.4±1.0°, *p*<0.001). At toe off, the obese knees were in a less flexed position than in the control group (27.6±9.7° vs. 36.1±3.2°, *p* = 0.024, Fig 3A). The total range of the flexion-extension motion was less in the group with obesity than in the control group (27.5±9.1° vs. 39.9±3.1°, *p* = 0.002). In knee adduction-abduction, the obese group had a similar range of motion compared to the control group (3.2±1.6° vs. 3.0±0.3°, *p* = 0.626), but at toe off, the obese knees were in a more adducted position compared to the control group (-3.2±1.2° vs. -5.8±0.9°, *p*<0.001, Fig 3B). In axial rotation, the obese knees did not show a significant difference compared to the controls (Fig 3C), and the range of internal-external rotation was not significantly different (6.7±3.6° vs. 9.2±2.1°, *p* = 0.082).

The tibial position with respect to femur in the obese group was more anterior than in the healthy group during most of the stance phase. Specifically, the obese knees were in more anterior translation than the control group at initial contact (1.5±4.1mm vs. -2.6±0.7mm, *p* = 0.011, Fig 3E), at the end of the mid-stance phase (0.8±2.7mm vs. -4.0±0.5mm, *p*<0.001), and at the end of terminal stance (1.4±2.0 mm vs. -2.9±0.7mm, *p*<0.001), but the translation at the end of the stance phase was not significantly different (3.1±2.6mm vs. 2.3±1.0mm, *p* = 0.395). Also, the range of anterior-posterior translation was not significantly different in the groups (2.5±1.0 mm vs. 6.4±1.2 mm, *p* = 0.151) (Fig 3E). The obese group had less range of medial-lateral translation than the control group (2.7±1.1mm vs. 4.3±0.6mm, *p* = 0.001), and were significantly different at the end of loading response and terminal stance phases (Fig 3D). The obese knees did not have a significantly different range of superior-inferior translation (1.8±0.6mm vs. 2.8±1.1mm, *p* = 0.054), but had significantly lower values than the control group during most of the stance phase of the gait cycle (Fig 3F).

## Discussion

This study investigated individuals with obesity and knee pain in standing and during gait under DFIS surveillance. While standing, the knee was in about 7.4° hyperextension, slight abduction (valgus), and about 5.6° of external rotation. During treadmill gait, the largest rotational excursion was in flexion-extension and the largest translational excursion was in the anterior-posterior direction, while motions in the other planes were smaller.

Few studies have evaluated standing posture in individuals with obesity. One study using a radiographic hip-knee-ankle measurement, found that obese individuals stood in a slightly knee flexed position [19]. However our obese individuals with knee pain tended to use a hyperextension strategy in standing. The adaptation in standing posture may be due to the high BMI in our sample (≥ 35) and knee pain. This posture is thought to reduce the demand on the quadriceps and could potentially prevent fatigue [30]. Our study also provided information in the other degrees of freedom. The analysis of the relative position between the femur and tibia indicated that the center of the femur was near the center in anterior-posterior direction, but located at the medial portion of the tibial plateau. These data provide additional data for more complete understanding of standing posture correction.

During walking, spatiotemporal parameters may be affected by gait speed. The speed we tested was controlled at 0.67m/s (1.5 mph), which is slightly slower than the reported self-selected walking speed of 0.73 to 1.08 m/s for an obese population [7, 31]. At this gait speed, we found that obese individuals with knee pain had slightly increased cadence and decreased stride length compared to individuals walking at a similar gait speed [7, 32], and had increased stride duration and stance phase compared to individuals walking at a faster gait speed [9, 31]. The speed preference and participant characteristics, such as knee pain and presence of osteoarthritis, may contribute to differences between studies.

Several studies have reported knee kinematics during gait using skin marker motion analysis in obese individuals [5, 6, 8]. In these studies, obese individuals were found to walk with a similar pattern or with more knee extension and reduced range of motion in the sagittal plane compared to non-obese individuals, and this strategy was assumed to decrease the exertion of the knee extensors and prevent fatigue [10, 33]. Our findings were not in full agreement with past studies [5, 8]. Similar to past studies, our obese individuals walked with a smaller range of flexion-extension motion during the stance phase compared to a healthy population (Fig 3A) [22]. However, our obese individuals walked in a more flexed knee position. While it is commonly thought that a more knee extended position will reduce demand on muscle, our participants did not display this adaptation. The combined smaller range of flexion-extension motion with a more knee flexed position during walking may increase the demands on the quadriceps and other extensors, and this gait pattern may result in early muscle fatigue. Our findings also showed a larger variation in knee flexion angles at initial contact, indicating different gait adaptation strategies could be used among individuals. The initial contact flexion angles were weakly correlated with their WOMAC pain score during walking (r = -0.34), suggesting that pain status may be a major contributor to the gait pattern.

Knee pain is a commonly reported symptom by patients with knee OA, and kinematic changes in the sagittal plane during gait were frequently reported [12–14]. Individuals with knee OA walked in a more extended position and reduced range of flexion-extension motion. This gait pattern had been further replicated by studies of experimental knee pain [34]. Obese individuals have also been found to walk with a similar gait pattern. Our study including individuals with combined obesity and knee pain walked with a smaller range of flexion-extension motion similar to patients with knee OA, but in a more knee flexed position during most stance phase when compared to a healthy non-obese group. This finding suggests that our obese individuals with knee pain may adopt a unique gait pattern, which is not typically found in patients with either obesity or knee OA.

A previous study found no difference in axial rotation between obese and non-obese groups [6], and our findings were in agreement with this; however, the pattern of axial rotation in our participants seemed to differ from that of healthy knees [22]. Healthy knees internally rotate after initial contact to a peak rotation at the end of loading response (Fig 3C) [22]. Instead, the tibia in our obese individuals was externally rotating to a peak of about 3.9° around the end of mid-stance and reversed after that point, implying the pivoting mechanism observed in healthy participants [22] may be changed in obese individuals. The large variation in the internal-external rotation angles in our obese individuals suggests that obese individuals may adopt diverse strategies to avoid pain during walking.

In the coronal plane, past studies found similar or more adduction in obese individuals compared to non-obese group during gait [5, 6]. In our study, we found adduction-abduction in coronal plane motion in our obese group was not significantly different compared to our control group (Fig 3B) [22]. We note that the majority of our subjects did not have medial osteoarthritis which might have been present in previous studies.

The smaller range of motion of the obese patients indicated that these individuals use a stiffening knee strategy despite maintaining the knee in more flexion during functional activities [35]. This strategy for reduced range of motion is also found in knee OA patients [12–14, 36] and obese individuals without knee pain [8], meaning both extra body weight and pain contribute to the gait pattern change. As walking has been routinely suggested for obese patients as a safe activity to increase energy expenditure, increasing the knee joint range of motion while walking should be addressed, and this could potentially better distribute contact stress to prevent the local stress concentration.

Several limitations need to be mentioned in interpreting the results. First, our participants were severely obese (BMI ≥ 35.0) and with knee pain; therefore, the results may not generalize to the less obese population (BMI: 30.0–34.9) with or without knee pain. Our study sample was 80% female, so the results may not generalize to all obese individuals with knee pain, although, like our sample, most obese individuals with knee pain are women. The control group was predominantly male, and this may also have contributed to the differences in kinematics found. Treadmill walking was used in this study to assess the kinematics, so the results may not generalize to walking overground. The definition of the coordinate system in this study was based on local bone geometry; therefore, the values found in this study may be slightly different from studies using other coordinate system definitions. Lastly the loading from the lead protection gowns (9.1kg, 7.0–10.6% of body mass of the obese participants) could have contributed to the gait pattern changes; however, it is unlikely that this contributed significantly to the differences found.

In conclusion, obese individuals with knee pain used hyper-extension knee posture while standing, but maintained the knee in more flexion during gait with reduced overall range of motion in the 6DOF analysis compared to a healthy group. In addition to the facilitation of greater total range of sagittal plane motion with a knee extension strategy during gait, increasing the knee joint range of motion in other directions should be addressed in weight management exercise programs.

## Supporting information

S1 File Gait data file. Six degree-of-freedom kinematics during stance phase of treadmill gait in obese individuals with knee pain.
(CSV)

## References

[1] Fryar CD, Carroll MD, Ogden CL. Prevalence of Overweight, Obesity, and Extreme Obesity Among Adults: United States, 1960–1962 Through 2011–2012. NCHS Health E-Stat [Web Page]. 2014 [cited 2016 July 28]. http://www.cdc.gov/nchs/data/hestat/obesity_adult_11_12/obesity_adult_11_12.htm.

[2] World Health Organization. World Health Statistics Report 2012 [Web Page]. [cited 2016 July 28]. http://apps.who.int/iris/bitstream/10665/44844/1/9789241564441_eng.pdf.

[3] Ogden CL, Carroll MD, Kit BK, Flegal KM. Prevalence of childhood and adult obesity in the United States, 2011–2012. JAMA. 2014;311(8):806–14. Epub 2014/02/27. 10.1001/jama.2014.732 24570244 PMC4770258

[4] Sturmer T, Gunther KP, Brenner H. Obesity, overweight and patterns of osteoarthritis: the Ulm Osteoarthritis Study. J Clin Epidemiol. 2000;53(3):307–13. Epub 2000/04/13. 10760642 10.1016/s0895-4356(99)00162-6

[5] Ko S, Stenholm S, Ferrucci L. Characteristic gait patterns in older adults with obesity—results from the Baltimore Longitudinal Study of Aging. Journal of biomechanics. 2010;43(6):1104–10. Epub 2010/01/19. 10.1016/j.jbiomech.2009.12.004 20080238 PMC2849896

[6] Lai PP, Leung AK, Li AN, Zhang M. Three-dimensional gait analysis of obese adults. Clinical biomechanics (Bristol, Avon). 2008;23 Suppl 1:S2–6. Epub 2008/04/01. 10.1016/j.clinbiomech.2008.02.004 18374462

[7] de Souza SA, Faintuch J, Valezi AC, Sant' Anna AF, Gama-Rodrigues JJ, de Batista Fonseca IC, et al Gait cinematic analysis in morbidly obese patients. Obes Surg. 2005;15(9):1238–42. Epub 2005/11/02. 10.1381/096089205774512627 16259878

[8] Haight DJ, Lerner ZF, Board WJ, Browning RC. A comparison of slow, uphill and fast, level walking on lower extremity biomechanics and tibiofemoral joint loading in obese and nonobese adults. Journal of orthopaedic research: official publication of the Orthopaedic Research Society. 2014;32(2):324–30. Epub 2013/10/16. 24127395 10.1002/jor.22497

[9] Sheehan KJ, Gormley J. The influence of excess body mass on adult gait. Clinical biomechanics (Bristol, Avon). 2013;28(3):337–43. Epub 2013/02/06. 10.1016/j.clinbiomech.2013.01.007 23380662

[10] McMillan AG, Pulver AM, Collier DN, Williams DS. Sagittal and frontal plane joint mechanics throughout the stance phase of walking in adolescents who are obese. Gait & posture. 2010;32(2):263–8. Epub 2010/06/25. 20573511 10.1016/j.gaitpost.2010.05.008

[11] Vismara L, Romei M, Galli M, Montesano A, Baccalaro G, Crivellini M, et al Clinical implications of gait analysis in the rehabilitation of adult patients with "Prader-Willi" Syndrome: a cross-sectional comparative study ("Prader-Willi" Syndrome vs matched obese patients and healthy subjects). Journal of neuroengineering and rehabilitation. 2007;4:14 10.1186/1743-0003-4-14 17493259 PMC1872029

[12] Al-Zahrani KS, Bakheit AM. A study of the gait characteristics of patients with chronic osteoarthritis of the knee. Disability and rehabilitation. 2002;24(5):275–80. 12004973 10.1080/09638280110087098

[13] Baliunas AJ, Hurwitz DE, Ryals AB, Karrar A, Case JP, Block JA, et al Increased knee joint loads during walking are present in subjects with knee osteoarthritis. Osteoarthritis and cartilage / OARS, Osteoarthritis Research Society. 2002;10(7):573–9. 10.1053/joca.2002.0797 12127838

[14] Kaufman KR, Hughes C, Morrey BF, Morrey M, An KN. Gait characteristics of patients with knee osteoarthritis. Journal of biomechanics. 2001;34(7):907–15. 11410174 10.1016/s0021-9290(01)00036-7

[15] Lerner ZF, Board WJ, Browning RC. Effects of an obesity-specific marker set on estimated muscle and joint forces in walking. Medicine and science in sports and exercise. 2014;46(6):1261–7. Epub 2014/02/13. 10.1249/MSS.0000000000000218 24518193

[16] Peters A, Galna B, Sangeux M, Morris M, Baker R. Quantification of soft tissue artifact in lower limb human motion analysis: a systematic review. Gait & posture. 2010;31(1):1–8. Epub 2009/10/27. 19853455 10.1016/j.gaitpost.2009.09.004

[17] Tsai TY, Lu TW, Kuo MY, Lin CC. Effects of soft tissue artifacts on the calculated kinematics and kinetics of the knee during stair-ascent. Journal of biomechanics. 2011;44(6):1182–8. Epub 2011/02/08. 10.1016/j.jbiomech.2011.01.009 21296352

[18] Cappozzo A, Catani F, Leardini A, Benedetti MG, Croce UD. Position and orientation in space of bones during movement: experimental artefacts. Clinical biomechanics (Bristol, Avon). 1996;11(2):90–100. Epub 1996/03/01. 10.1016/0268-0033(95)00046-1 11415604

[19] Jalai CM, Diebo BG, Cruz DL, Poorman GW, Vira S, Buckland AJ, et al The impact of obesity on compensatory mechanisms in response to progressive sagittal malalignment. The spine journal: official journal of the North American Spine Society. 2016. 10.1016/j.spinee.2016.11.016 27916684

[20] Messier SP. Diet and exercise for obese adults with knee osteoarthritis. Clin Geriatr Med. 2010;26(3):461–77. Epub 2010/08/12. 10.1016/j.cger.2010.05.001 20699166 PMC3444812

[21] Poirier P, Despres JP. Exercise in weight management of obesity. Cardiol Clin. 2001;19(3):459–70. Epub 2001/09/26. 11570117 10.1016/s0733-8651(05)70229-0

[22] Kozanek M, Hosseini A, Liu F, Van de Velde SK, Gill TJ, Rubash HE, et al Tibiofemoral kinematics and condylar motion during the stance phase of gait. Journal of biomechanics. 2009;42(12):1877–84. Epub 2009/06/06. 10.1016/j.jbiomech.2009.05.003 19497573 PMC2725209

[23] Li JS, Hosseini A, Cancre L, Ryan N, Rubash HE, Li G. Kinematic characteristics of the tibiofemoral joint during a step-up activity. Gait & posture. 2013;38(4):712–6. Epub 2013/04/02. 23541765 10.1016/j.gaitpost.2013.03.004 PMC3722253

[24] Tsai TY, Li JS, Wang S, Lin H, Malchau H, Li G, et al A novel dual fluoroscopic imaging method for determination of THA kinematics: in-vitro and in-vivo study. J Biomech. 2013;46(7):1300–4. Epub 2013/03/19. 10.1016/j.jbiomech.2013.02.010 23497800

[25] Li G, Van de Velde SK, Bingham JT. Validation of a non-invasive fluoroscopic imaging technique for the measurement of dynamic knee joint motion. J Biomech. 2008;41(7):1616–22. Epub 2008/04/09. 10.1016/j.jbiomech.2008.01.034 18394629

[26] Kellgren JH, Lawrence JS. Radiological assessment of osteo-arthrosis. Annals of the rheumatic diseases. 1957;16(4):494–502. Epub 1957/12/01. 13498604 10.1136/ard.16.4.494 PMC1006995

[27] Gronenschild E. Correction for geometric image distortion in the x-ray imaging chain: local technique versus global technique. Medical physics. 1999;26(12):2602–16. 10.1118/1.598800 10619246

[28] Gronenschild E. The accuracy and reproducibility of a global method to correct for geometric image distortion in the x-ray imaging chain. Medical physics. 1997;24(12):1875–88. 10.1118/1.598101 9434970

[29] Grood ES, Suntay WJ. A joint coordinate system for the clinical description of three-dimensional motions: application to the knee. J Biomech Eng. 1983;105(2):136–44. Epub 1983/05/01. 6865355 10.1115/1.3138397

[30] Loudon JK, Goist HL, Loudon KL. Genu recurvatum syndrome. The Journal of orthopaedic and sports physical therapy. 1998;27(5):361–7. 10.2519/jospt.1998.27.5.361 9580896

[31] Blaszczyk JW, Plewa M, Cieslinska-Swider J, Bacik B, Zahorska-Markiewicz B, Markiewicz A. Impact of excess body weight on walking at the preferred speed. Acta Neurobiol Exp (Wars). 2011;71(4):528–40. Epub 2012/01/13. 22237498 10.55782/ane-2011-1869

[32] Browning RC, Kram R. Effects of obesity on the biomechanics of walking at different speeds. Medicine and science in sports and exercise. 2007;39(9):1632–41. Epub 2007/09/07. 10.1249/mss.0b013e318076b54b 17805097

[33] Maffiuletti NA, Jubeau M, Munzinger U, Bizzini M, Agosti F, De Col A, et al Differences in quadriceps muscle strength and fatigue between lean and obese subjects. Eur J Appl Physiol. 2007;101(1):51–9. Epub 2007/05/04. 10.1007/s00421-007-0471-2 17476522

[34] Henriksen M, Graven-Nielsen T, Aaboe J, Andriacchi TP, Bliddal H. Gait changes in patients with knee osteoarthritis are replicated by experimental knee pain. Arthritis Care Res (Hoboken). 2010;62(4):501–9. Epub 2010/04/15. 20391505 10.1002/acr.20033

[35] Bytyqi D, Shabani B, Lustig S, Cheze L, Karahoda Gjurgjeala N, Neyret P. Gait knee kinematic alterations in medial osteoarthritis: three dimensional assessment. Int Orthop. 2014;38(6):1191–8. Epub 2014/03/13. 10.1007/s00264-014-2312-3 24619388 PMC4037512

[36] Duffell LD, Southgate DF, Gulati V, McGregor AH. Balance and gait adaptations in patients with early knee osteoarthritis. Gait & posture. 2014;39(4):1057–61. 24582072 10.1016/j.gaitpost.2014.01.005 PMC3989045

