# Correction: Six degree-of-freedom knee joint kinematics in obese individuals with knee pain during gait

**DOI:** 10.1371/journal.pone.0213084

**Published:** 2019-02-22

**Authors:** Jing-Sheng Li, Tsung-Yuan Tsai, David T. Felson, Guoan Li, Cara L. Lewis

There is an error in the data for Fig 3D and 3E, which use control group data from a previously published paper [2].

The translation in the anterior-posterior direction and medial-lateral direction among the control group is presented as femur relative to tibia in the article by Kozanek et al. (2009) [2] but not converted to the data of tibia relative to femur, the way the authors of the *PLOS ONE* article define these measures for the obese population. As the description of the knee motion in the two studies is not the same, the authors converted the kinematic data of the control group (Kozanek's data) to the definition of the data published in *PLOS ONE*. Unfortunately, some data in the control group were not converted correctly. The error affects two of the six comparisons of the data between the obese group and the control group (Fig 3D and 3E) but does not affect the conclusions or overall scientific understanding of the study. As such, the penultimate sentence of the Abstract is incorrect. The correct sentence is: Specifically, obese individuals with knee pain maintained the knee in more flexion with a reduced total range of knee flexion and had a different pattern in the anterior-posterior translation during most of the stance phase of the gait cycle.

The last paragraph of the Results section is also incorrect. The correct paragraph is: The range of anterior-posterior translation in the obese group was not significantly different from in the healthy group (5.1±2.5 mm vs. 6.4±1.2 mm, *p* = 0.151) (Fig 3E); however, the tibial position with respect to femur was different between the two groups in the second half of the stance phase. Specifically, the obese knees were in a more posterior position than the control group at the end of the mid-stance phase (0.8±2.7 mm vs. 4.0±0.5 mm, *p* = 0.005), and at the end of terminal stance phase (1.4±2.0 mm vs. 2.9±0.7 mm, *p* = 0.044), but in a more anterior position than the control group at the end of the stance phase (3.1±2.6 mm vs. -2.3±1.0 mm, *p*<0.001). The obese group had less range of medial-lateral translation than the control group (2.7±1.1 mm vs. 4.3±0.6 mm, *p* = 0.001), and were consistently in a more lateral position than the control group throughout the stance phase of the gait cycle (Fig 3D). The obese knees did not have a significantly different range of superior-inferior translation (1.8±0.6 mm vs. 2.8±1.1 mm, *p* = 0.054), but had significantly lower values than the control group during most of the stance phase of the gait cycle (Fig 3F).

Additionally, Fig 3 is incorrect. The authors have provided a corrected version here.

**Fig 3:**
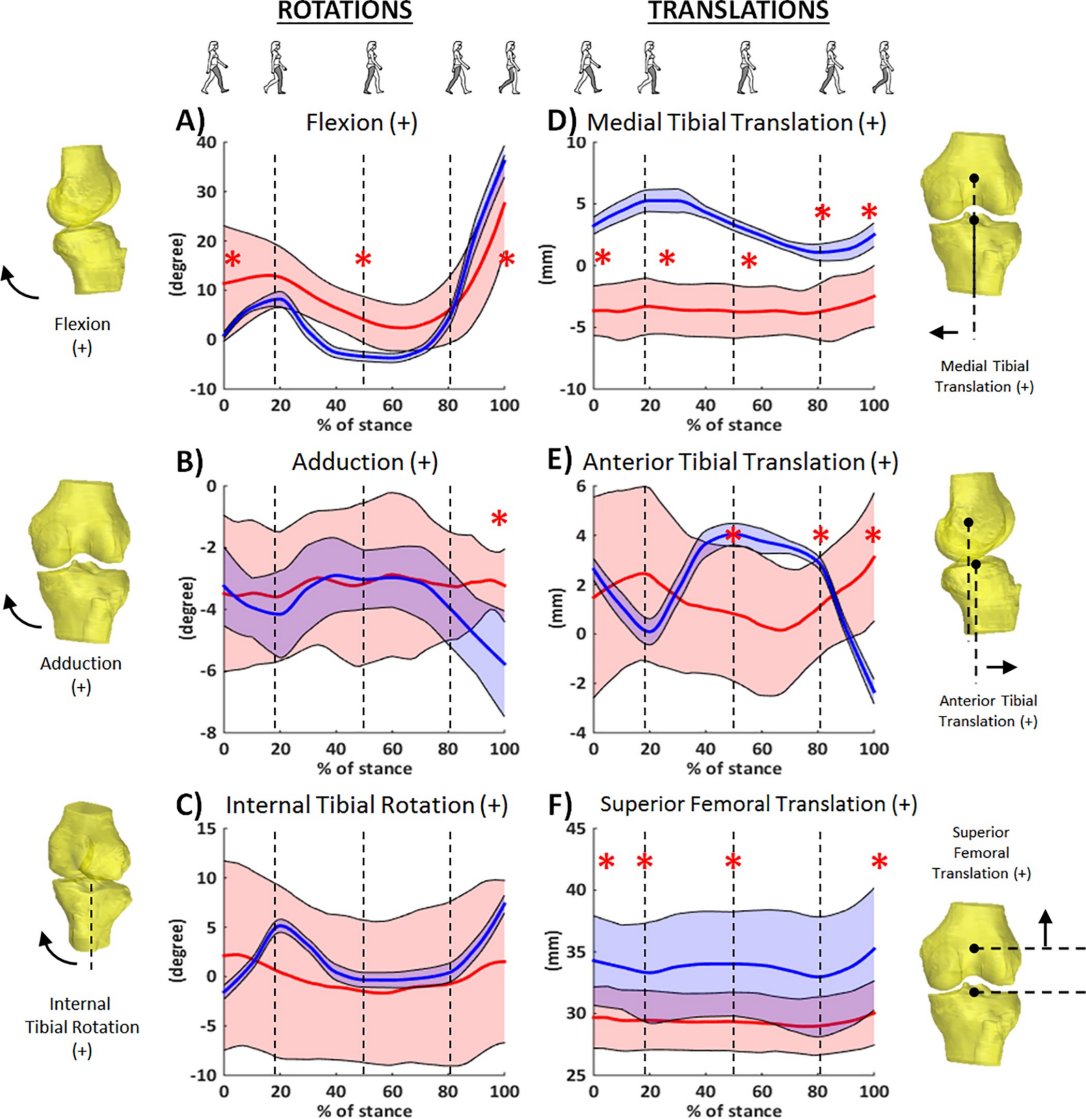
Six degree-of-freedom kinematics during stance phase of treadmill gait in obese individuals with knee pain (red. S1 File) and a healthy population (blue, data previously published in Kozanek et al. J Biomech. 2009;42(12):1877–1884.). Solid line indicates the mean and shade area for ±1SD. Asterisk denotes significant difference between two groups.

Please note that for the ranges of motion presented above and in the text of the Results section, values for the specific range were calculated first for each subject, and then the average of those subject-specific values is presented. By contrast, the graph presented in Fig 3 is plotted using the group means of the time series data. Due to variability between subjects, the peaks (and subsequently the range) are attenuated when plotted in this manner.

The original unconverted control group dataset was obtained from a previous study [2]; the converted control group dataset (tibia relative to femur) is included here as a Supporting Information file, S1 File, with permission.

## Supporting information

S1 File Gait data file, control group. Six degree-of-freedom kinematics during stance phase of treadmill gait in healthy individuals with knee pain.
(CSV)
